# Role of psychological resilience and psychological distress in linking fear of disease progression to quality of life in chronic heart failure: a cross-sectional serial mediation analysis

**DOI:** 10.3389/fpsyt.2026.1809638

**Published:** 2026-05-25

**Authors:** Hong Ding, Xiaoxia Fang, Shixun Li, Liyun Miao, Xiao Wu

**Affiliations:** Department of Cardiology Ward II, Xinxiang Central Hospital, Xinxiang, Henan, China

**Keywords:** chronic heart failure, fear of disease progression, psychological distress, quality of life, resilience

## Abstract

**Objective:**

To examine whether psychological resilience and psychological distress serially mediate the association between fear of disease progression and quality of life (QoL) in patients with chronic heart failure (CHF).

**Methods:**

This cross-sectional study enrolled 212 patients with CHF admitted between June 2023 and June 2025. Assessment tools included a demographic questionnaire, the Fear of Progression Questionnaire (FoP-Q), the Connor–Davidson Resilience Scale (CD-RISC), the Depression Anxiety Stress Scales-21 (DASS-21), and the Minnesota Living with Heart Failure Questionnaire (MLHFQ). Correlation and serial mediation analyses were performed using IBM SPSS Statistics for Windows, version 22.0, and the PROCESS macro, with the bootstrap method (5,000 resamples) used to test the mediation effects.

**Results:**

The mean scores were 43.60 ± 8.32 for FoP-Q, 52.71 ± 14.28 for CD-RISC, 44.29 ± 10.68 for DASS-21, and 48.63 ± 10.85 for MLHFQ. Correlation analysis indicated that FoP was negatively correlated with psychological resilience (r = −0.775) and positively correlated with psychological distress and MLHFQ scores (r = 0.868 and 0.773, respectively; all P < 0.05). Psychological resilience was negatively correlated with both psychological distress and MLHFQ scores (r = −0.728 and −0.744, respectively), while psychological distress was positively correlated with MLHFQ scores (r = 0.745; all P < 0.05). The mediation model revealed a direct effect of FoP on QoL (effect = 0.629, 41.14%), along with three indirect pathways: via psychological resilience alone (effect = 0.508, 33.22%), via psychological distress alone (effect = 0.344, 22.50%), and via the serial pathway from psychological resilience to psychological distress (effect = 0.048, 3.14%).

**Conclusion:**

Patients with CHF exhibited elevated levels of FoP and generally reduced QoL. Psychological resilience and psychological distress served as significant serial mediators in the relationship between FoP and QoL. FoP could directly reduce QoL in patients with CHF and indirectly affect it by decreasing psychological resilience and exacerbating psychological distress. Clinical attention should be directed toward assessing the psychological status of patients with CHF, improving psychological resilience, alleviating negative emotions, reducing the adverse impact of FoP, and enhancing patients’ QoL.

## 1 Introduction

Chronic heart failure (CHF) is a condition that arises when a spectrum of cardiovascular disorders progresses to its terminal phase, characterized by impaired myocardial systolic or diastolic function, reduced exercise tolerance, and recurrent hospitalizations. Fear of progression (FoP), a common negative psychological reaction, refers to patients’ persistent concerns about disease deterioration, loss of function, death, and related impairment of social functioning. The incidence of FoP in the CHF population can reach 40%–60% (1). Studies have shown that FoP can reduce patients’ treatment compliance and aggravate negative emotions, thereby affecting quality of life (QoL) and increasing the risk of rehospitalization (2). The specific pathways by which FoP affects QoL among CHF patients have not yet been fully elucidated and require further exploration to guide clinical intervention.

Resilience, as a psychological trait that enables individuals to maintain psychological balance, actively adapt, and recover functioning when facing stress, trauma, or adversity, plays a protective role in patients with cardiovascular disease (3). Psychological resilience can improve QoL by regulating emotional responses, enhancing coping ability, and reducing the psychological burden related to disease (4). In the context of CHF, psychological distress is a common clinical phenomenon. Its core components include depression, anxiety, and stress, making it a predominant form of psychological morbidity in this patient population, with an incidence significantly higher than that in the general population (5). Psychological distress can aggravate myocardial injury by activating the neuroendocrine–immune axis, reduce patients’ self-management ability, and lead to a decline in QoL (6).

Based on stress and coping theory, FoP, as a chronic psychological stressor, may indirectly affect patients’ QoL by influencing their positive psychological resources (psychological resilience) and negative emotional states (psychological distress). Evidence suggests that psychological resilience is inversely associated with subsequent levels of psychological distress (7). Within the CHF population, it has not been sufficiently examined whether psychological resilience and psychological distress serve as sequential mediators linking FoP to QoL. To address this gap, the present study examined the chain-mediation role of these psychological factors between FoP and QoL.

## 2 Data and methods

This was a single-center cross-sectional study conducted in the Department of Cardiology Ward II of Xinxiang Central Hospital, a tertiary grade A general hospital located in Xinxiang City, Henan Province, China, from June 2023 to June 2025. All participants were consecutively enrolled from the inpatient department of the aforementioned ward during the study period, and no convenience sampling was used. The study was designed to explore the correlations and mediating relationships among fear of disease progression, psychological resilience, psychological distress, and QoL in patients with CHF at a specific time point without follow-up.

### 2.1 Research object

All eligible patients admitted during the study period were screened and included in the order of admission, with no subjective selection, pre-set enrollment quota, or exclusion of eligible patients beyond the pre-specified inclusion and exclusion criteria. The inclusion criteria were as follows: (1) a confirmed diagnosis of CHF according to standard criteria (8), with left ventricular ejection fraction (LVEF) ≤ 50% verified through echocardiographic examination within the past month and a NYHA cardiac function classification of II–IV; (2) age ≥ 18 years; (3) disease duration ≥ 6 months, with a clear history of diagnosis and treatment of CHF and stable condition; (4) clear consciousness, basic reading comprehension ability, and the ability to understand and complete the questionnaire independently or with the assistance of researchers; and (5) provision of written informed consent to participate. The exclusion criteria were as follows: (1) patients with severe liver or renal failure, malignant tumors (at the stage of radiotherapy and chemotherapy or late metastasis), severe cognitive impairment, schizophrenia/bipolar disorder, severe lung disease, autoimmune disease, or other diseases that may interfere with the evaluation of mental state and QoL; (2) those with serious language communication barriers (such as aphasia or dysarthria), moderate to severe hearing impairment (unable to communicate normally through hearing aids), visual impairment (unable to recognize questionnaire text), or physical limitations that prevent completion of the questionnaire; (3) those in the acute phase of heart failure or with acute complications such as acute myocardial infarction, severe arrhythmia, or pulmonary infection within the past week, and whose vital signs (blood pressure, heart rate) were not effectively controlled; (4) those who had experienced major life events (such as death of relatives, major trauma, or unemployment) within the past three months, or were undergoing professional psychotherapy or psychiatric medication adjustment, which may affect the evaluation of psychological resilience and psychological distress; and (5) individuals with a history of drug abuse, alcohol dependence, or poor compliance who were unable to cooperate with the questionnaire survey and data collection.

According to the classical sample size requirement for mediation analysis proposed by Hayes et al., the minimum sample size for a serial mediation model should be 10 times the number of core path coefficients in the model to ensure the stability of the effect estimation. The serial mediation model constructed in this study contained six core path coefficients; therefore, the minimum required sample size was 60. The final sample size of 212 far exceeded this minimum requirement, ensuring the robustness of the mediation effect test.

*A priori* power analysis was performed using G*Power 3.1 software prior to study initiation. For the multiple linear regression–based mediation analysis, we set the two-tailed test level α = 0.05, power (1 − β) = 0.95, and expected effect size f² = 0.15 (moderate effect size, based on previously published similar studies in CHF populations). The results indicated that the minimum required sample size was 138. Considering a possible 15% invalid questionnaire rate, we set the pre-study target minimum sample size at 160. The final enrolled sample size of 212 fully met this requirement, with an actual test power of 0.98, which effectively controlled Type I and Type II errors and ensured the reliability of the statistical results.

### 2.2 Data collection instruments

#### 2.2.1 Participant information sheet

Designed by the researchers, this questionnaire collected two categories of data: (1) demographic and sociological data, including age, gender, educational level, and marital status; and (2) clinical disease-related data, including disease duration and NYHA cardiac function classification. Based on the univariate analysis results of this study and previously published evidence confirming their independent influence on the QoL of patients with CHF, age, disease duration, and NYHA cardiac function classification were ultimately selected as covariates to be controlled in the serial mediation model to eliminate confounding effects of these variables on the association between the core research variables.

#### 2.2.2 FoP-Q scale

This scale was designed to assess patients’ fear of disease progression (9). It consists of 12 items, each rated on a 5-point Likert scale (1 = “not at all worried” to 5 = “extremely worried”). The total score ranges from 12 to 60, with higher scores indicating greater severity of FoP. The scale demonstrated good reliability, with a Cronbach’s alpha of 0.89 and a test–retest reliability of 0.83.

#### 2.2.3 Minnesota living with heart failure questionnaire

Patient QoL was evaluated using the MLHFQ (10). This instrument was specifically developed to assess the physical functioning, emotional state, and social adaptability of patients with heart failure. It contains 21 items, structured into three domains: physical (8 items), emotional (5 items), and other (8 items). Each item is scored on a 0–5 Likert scale (0 = no impact at all, 5 = extreme impact), with a total score ranging from 0 to 105. Higher scores indicate a greater impact of heart failure on patients’ QoL, that is, poorer QoL.

#### 2.2.4 Connor Davidson resilience scale (11)

The CD-RISC is structured to assess resilience across three dimensions—hardiness, strength, and optimism—and comprises 25 items. It uses a 5-point Likert scale (1 = completely untrue, 5 = completely true), with a total score ranging from 25 to 125. Higher scores indicate greater psychological resilience.

#### 2.2.5 Depression anxiety stress scales -21 items

The Chinese version of the DASS-21 scale (12), developed by Lovibond et al. and translated and revised by Chinese researchers, was used to assess patients’ psychological distress. It includes three dimensions—depression, anxiety, and stress—with a total of 21 items. A 0–3 Likert scale (0 = none at all, 3 = almost always) was used. The total score ranges from 0 to 63, with higher scores indicating more severe psychological distress.

### 2.3 Data collection

A questionnaire survey was conducted by uniformly trained researchers. Before the formal investigation, all researchers received standardized training covering the study purpose, questionnaire content, standardized non-inductive survey language, on-site quality control procedures, and data confidentiality principles. The consistency of survey implementation among all researchers was verified through a pre-investigation assessment to ensure standardization of the survey process and minimize information bias.

Before the survey, the purpose, content, process, and confidentiality principles of the study were explained in detail to the patients, and the questionnaire was administered after obtaining informed consent. Patients with a higher educational level who were able to complete the questionnaire independently were instructed to do so. For patients with a lower educational level or those unable to complete the questionnaire independently, the researcher read the items aloud in a non-inductive manner and recorded responses based on the patients’ answers. After completion of the questionnaire, the researcher checked it on-site to ensure completeness and absence of omissions. For questionnaires completed irregularly, patients were asked to supplement and correct them in a timely manner, and valid questionnaires were collected and coded.

### 2.4 Statistical analysis

The collected data were processed and analyzed using the statistical software SPSS (version 22.0). Normality of continuous data was assessed using the Shapiro–Wilk test. Data conforming to a normal distribution are presented as (x̄ ± s) and were analyzed accordingly. An independent samples t-test was applied for two-group comparisons. For multi-group comparisons, data were analyzed using one-way ANOVA, with *post hoc* Bonferroni tests for pairwise comparisons. Count data were expressed as n (%). Correlations among FoP, psychological resilience, psychological distress, and QoL in patients with CHF were analyzed using Pearson ‘scorrelation analysis.

The serial mediation effect was tested using the PROCESS macro (Model 6) developed by Hayes for SPSS, with FoP as the independent variable, QoL as the dependent variable, psychological resilience and psychological distress as serial mediators, and age, disease duration, and NYHA cardiac function classification as covariates to control confounding effects. The bias-corrected bootstrap method with 5000 resamples was used to verify the significance of the mediation effects. A 95% confidence interval (CI) that did not include 0 was considered indicative of a statistically significant mediation effect.

Before formal statistical analysis, the missing rates of all variables were systematically assessed. A pre-specified hierarchical processing strategy was adopted: for variables with a missing rate < 5%, complete-case analysis was used; for variables with a missing rate ≥ 5%, multiple imputation (MI) with 20 imputed datasets was performed to ensure the stability and reliability of the statistical results. Statistical significance was set at p < 0.05.

## 3 Results

### 3.1 Characteristics of the study participants

#### 3.1.1 Participant flow

During the prespecified study period (June 2023 to June 2025), 258 CHF inpatients admitted to the study ward were initially screened for eligibility in strict accordance with the consecutive sampling strategy. The detailed participant flow and screening process are as follows: 12 patients were excluded for not meeting the prespecified inclusion criteria (8 patients with LVEF > 50%, 4 patients with disease duration < 6 months); 18 patients were excluded for meeting the exclusion criteria (6 patients with severe combined organ dysfunction or malignant tumors, 5 patients with acute heart failure exacerbation or acute complications, 4 patients with severe cognitive impairment or mental illness, and 3 patients with major adverse life events in the past 3 months). Among the remaining 228 eligible patients, 10 patients refused to participate in the study (main reasons for non-participation: lack of time to complete the questionnaire, unwillingness to answer psychological-related questions, and concerns about privacy disclosure), and 6 patients were unable to complete the questionnaire due to severe communication or visual barriers. Finally, 212 eligible patients were formally enrolled, with an effective enrollment rate of 82.17% (212/258) and a 100% valid questionnaire response rate among all enrolled participants.

#### 3.1.2 Baseline characteristics of enrolled participants

Among the 212 patients with CHF, 120 (56.60%) were male and 92 (43.40%) were women. The mean age was 61.89 ± 9.23 years (range, 40–80 years). Regarding educational level, 46 patients (21.70%) had primary school education or below, 84 (39.62%) had completed junior high school, 50 (23.58%) had high school or technical secondary education, and 32 (15.09%) had a college degree or above, with junior high school being the most common level. In terms of marital status, 176 patients were married (83.02%), while 36 were unmarried, divorced, or widowed (16.98%). For NYHA cardiac function classification, 68 cases (32.08%) were grade II, 109 cases (51.42%) were grade III, and 35 cases (16.51%) were grade IV.

### 3.2 Scores of each scale

The MLHFQ revealed a total score of (48.63 ± 10.85) in the 212 patients with CHF, including (20.35 ± 5.76) for the physical domain, (14.82 ± 4.31) for the emotional domain, and (13.46 ± 4.12) for other domains. The total score of the FoP-Q was (43.60 ± 8.32). The total score of the CD-RISC was (52.71 ± 14.28), including tenacity (20.12 ± 5.34), strength (18.45 ± 4.89), and optimism (14.14 ± 3.95). The total score of the DASS-21 was (44.29 ± 10.68), including depression (16.83 ± 4.25), anxiety (15.72 ± 3.98), and stress (11.74 ± 3.86). Details are shown in **Table 1**.

**Table 1:** Score of each scale (x̄ ± s).

Item	Dimension	Score
FoP-Q	Total score	43.60 ± 8.32
MLHFQ	Total score	48.63 ± 10.85
	Physical	20.35 ± 5.76
	Emotional	14.82 ± 4.31
	Other	13.46 ± 4.12
CD-RISC	Total score	52.71 ± 14.28
	Tenacity	20.12 ± 5.34
	Strength	18.45 ± 4.89
	Optimism	14.14 ± 3.95
DASS-21	Total score	44.29 ± 10.68
	Depression	16.83 ± 4.25
	Anxiety	15.72 ± 3.98
	Stress	11.74 ± 3.86

### 3.3 Comparison of QoL scores of CHF patients with different demographic and disease characteristics

Regarding age, significantly higher MLHFQ scores were observed in patients aged ≥ 60 years than in those aged < 60 years. Regarding disease duration, scores were also significantly higher in patients with a duration ≥ 18 months than in those with a duration < 18 months (P < 0.05). Furthermore, significant differences in MLHFQ scores were found across different NYHA functional classes in pairwise comparisons (P < 0.05). Specifically, patients in the NYHA class IV had significantly higher scores than those in both the NYHA classes II and III groups (P < 0.05). Details are shown in **Table 2**.

**Table 2:** Comparison of QoL scores of CHF patients with different demographic and disease characteristics (x̄ ± s).

Characteristic	Grouping	Number of cases	MLHFQ	*t/F*	*P*
Age	<60	89	43.89 ± 10.56	5.709	<0.001
	≥60	123	52.17 ± 10.32		
Gender	Male	120	47.53 ± 10.92	1.728	0.085
	Female	92	50.12 ± 10.68		
Educational level	Junior high school or below	130	49.37 ± 8.64	0.704	0.496
	Senior high school or technical secondary school	50	48.23 ± 10.72		
	College or above	32	47.39 ± 9.63		
Marital status	Married	176	47.85 ± 10.58	1.281	0.202
	Unmarried/divorced/widowed	36	50.36 ± 11.36		
NYHA cardiac function class	II	68	43.34 ± 8.92	12.096	<0.001
	III	109	47.96 ± 10.45^*^		
	IV	35	53.46 ± 10.79^*#^		
Disease duration (months)	<18	115	45.78 ± 10.74	4.265	<0.001
	≥18	97	52.01 ± 10.41		

^*^ indicates comparison with the NYHA class II, *P* < 0.05; ^#^indicates comparison with the NYHA class III, *P* < 0.05.

### 3.4 Correlation analysis between variables

Pearson correlation analyses are summarized as follows: FoP exhibited a strong negative correlation with psychological resilience (r = −0.775, P < 0.05) and was strongly and positively associated with both psychological distress and MLHFQ scores (r = 0.868 and 0.773, respectively; P < 0.05). Psychological resilience was inversely associated with psychological distress (r = −0.728, P < 0.05) and MLHFQ scores (r = −0.744, P < 0.05). MLHFQ scores exhibited a significant positive association with psychological distress (r = 0.745, P < 0.05). Details are shown in **Table 3**.

**Table 3:** Correlation analysis between variables.

Variable	1	2	3	4	5	6	7	8	9
1.FoP-Q	1	-0.775	-0.721	-0.683	-0.628	0.868	0.815	0.732	0.773
2.Total psychological resilience score	-0.775	1	0.852	0.817	0.763	-0.728	-0.694	-0.635	-0.744
3.Tenacity	-0.721	0.852	1	0.734	0.689	-0.687	-0.652	-0.598	-0.744
4.Strength	-0.683	0.817	0.734	1	0.721	-0.665	-0.631	-0.587	-0.712
5.Optimism	-0.628	0.763	0.689	0.721	1	-0.612	-0.593	-0.576	-0.685
6.Total psychological distress score	0.868	-0.728	-0.687	-0.665	-0.612	1	0.887	0.823	0.745
7.Depression	0.815	-0.694	-0.652	-0.631	-0.593	0.887	1	0.801	0.732
8.Anxiety	0.732	-0.635	-0.598	-0.587	-0.576	0.823	0.801	1	0.812
9.MLHFQ	0.773	-0.744	-0.744	-0.712	-0.685	0.745	0.732	0.715	1

Pearson correlation analysis, all *P* < 0.05.

### 3.5 Mediating effect model of resilience and psychological distress between FoP and QoL in patients with CHF

The FoP-Q total score (W = 0.982, P = 0.126), CD-RISC total score (W = 0.979, P = 0.087), DASS-21 total score (W = 0.984, P = 0.189), and MLHFQ total score (W = 0.980, P = 0.092) were assessed using the Shapiro–Wilk test. All P values were > 0.05, indicating that all core continuous variables conformed to a normal distribution. Meanwhile, the absolute values of skewness for all variables were < 1.2, and the absolute values of kurtosis were < 2.3, both well below the critical thresholds for non-normality. The normal Q–Q plots of all variables showed that the data points were closely distributed along the diagonal, and the histograms presented an approximately normal bell-shaped distribution, further verifying the normality of the data. There was no violation of the normality assumption in this study, which fully met the requirements for the application of the PROCESS macro and parametric tests, and no additional data transformation was required.

For all variables in the model, the variance inflation factor (VIF) values ranged from 1.018 to 2.213, all below the critical threshold of 10, and the tolerance values ranged from 0.452 to 0.982, all above the critical threshold of 0.1. In particular, for the regression equation with psychological resilience as the dependent variable, the VIF value of FoP was only 1.023 and the tolerance was 0.977, indicating that there was no multicollinearity between FoP and other covariates, and that the regression coefficient estimation was not affected by multicollinearity.

With FoP as the independent variable (X), QoL as the dependent variable (Y), and psychological resilience and psychological distress as serial mediating variables (M1, M2), and age, disease duration, and NYHA cardiac function classification controlled as covariates, the serial mediation model was constructed. The model showed that FoP had a positive predictive effect on QoL (β = 0.627), was a negative predictor of psychological resilience (β = −1.097), and was positively associated with psychological distress (β = 0.789). Psychological resilience was inversely associated with psychological distress in the regression model (β = −0.100). When both the independent and mediator variables were entered into the regression equation, two significant associations were observed: psychological resilience was a negative predictor of QoL (β = −0.463), whereas psychological distress was a positive predictor (β = 0.436). Details are shown in **Table 4**.

**Table 4:** Mediating effect model of resilience and psychological distress between FoP and QoL in patients with CHF.

Outcome variable	Predictive variable	Model coefficient			
		*β*	*SE*	*t*	*P*
QoL	FoP	0.627	0.177	3.550	<0.001
Psychological resilience	FoP	-1.097	0.062	-17.824	<0.001
Psychological distress	FoP	0.789	0.056	14.208	<0.001
Psychological distress	Psychological resilience	-0.100	0.039	-2.537	0.012
QoL	Psychological resilience	-0.463	0.091	-5.109	<0.001
	Psychological distress	0.436	0.157	2.772	0.006

### 3.6 The mediating effect of resilience and psychological distress between FoP and QoL in patients with CHF

**Figure 1:**
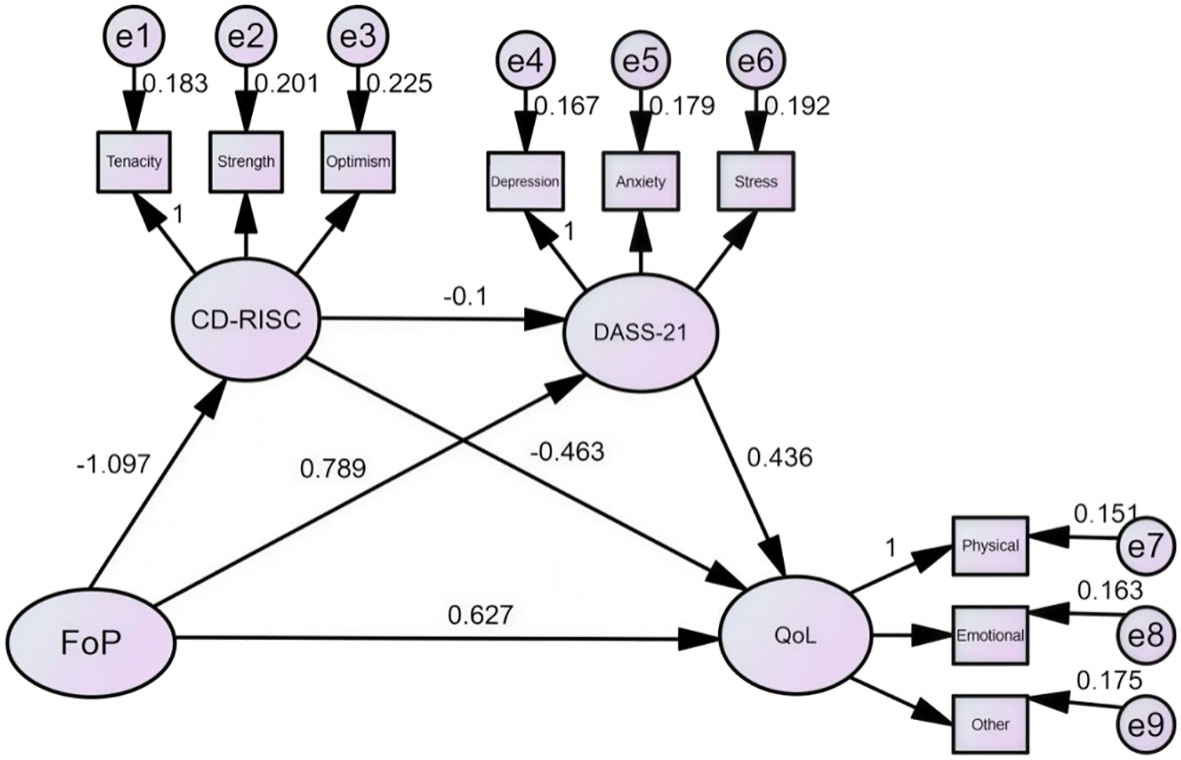
Serial mediation model illustrating the relationships between FoP, psychological resilience, psychological distress, and QoL in CHF patients.

After controlling for the confounding effects of age, disease duration, and NYHA cardiac function classification, FoP exerted both direct (β = 0.629, P = 41.14%) and indirect effects on QoL. The total indirect effect was mediated through three distinct pathways. The specific indirect effects were 0.508 (33.22%) via psychological resilience alone, 0.344 (22.50%) via psychological distress alone, and 0.048 (3.14%) via the sequential pathway from psychological resilience to psychological distress. This final pathway supports a chain mediation model. Details are presented in **Table 5** and **Figure 1**.

**Table 5:** The mediating effect of resilience and psychological distress between FoP and QoL in patients with CHF.

Effect	Effect path	Effect value	*SE*	*95%CI*	Effect ratio(%)
Total effect	FoP - QoL	1.529	0.087	1.358~1.699	100
Direct effect	FoP - QoL	0.629	0.177	0.280~0.978	41.14
Indirect effect	FoP - Psychological resilience - QoL	0.508	0.162	0.342~0.690	33.22
	FoP - Psychological distress - QoL	0.344	0.132	0.089~0.604	22.50
	FoP - Psychological resilience - Psychological distress - QoL	0.048	0.028	0.005~0.114	3.14

## 4 Discussion

The findings of the present study indicated that the average FoP score of the 212 patients with CHF was (43.60 ± 8.32), which was higher than that of healthy individuals and consistent with the conclusions of previous studies (13). Owing to the long disease course, recurrent illness, and uncertain prognosis, patients with CHF tend to continuously worry about disease progression, decline in functional ability, loss of self-care capacity, and the burden on their families. The average CD-RISC score was (52.71 ± 14.28), which was lower than that of the general domestic population (65.42 ± 13.90) (14), indicating that the overall level of psychological resilience in patients with CHF is relatively low. Long-term stress associated with chronic disease, repeated hospitalizations, and uncertainty about the future may weaken patients’ psychological adaptability, leading to reduced resilience. The average DASS-21 score was (44.29 ± 10.68), suggesting that most patients experienced significant psychological distress, which may be related to multiple stressors such as physical discomfort, psychological burden, and changes in social roles faced by patients with CHF, consistent with previous findings (15).

At the same time, the total MLHFQ score in this study was (48.63 ± 10.85). Further analysis revealed that patients aged ≥ 60 years, those with higher NYHA cardiac function classification, and those with longer disease duration had poorer QoL. Older patients are often accompanied by degenerative changes, reduced myocardial functional reserve, and comorbid conditions, such as hypertension and diabetes, which increase the cardiac burden. A higher NYHA classification reflects poorer cardiac pump function and lower activity tolerance, resulting in greater limitations in daily activities. A longer disease duration exposes patients to prolonged symptoms and repeated hospitalizations, leading to progressive impairment in physiological function, social functioning, and self-management ability, ultimately reducing QoL.

Our analysis confirmed a substantial direct negative effect of FoP on health-related QoL (HRQoL), accounting for over 40% of its total effect. This highlights FoP as a critical independent risk factor for diminished well-being in patients with CHF. As a chronic psychological stressor, FoP may influence QoL through psychophysiological interactions. It can induce persistent anxiety and fear, activate the hypothalamic–pituitary–adrenal axis, increase sympathetic nervous system activity, accelerate heart rate, elevate blood pressure, and increase myocardial oxygen consumption, thereby further impairing cardiac function and reducing physical functioning. In addition, FoP may lead patients to avoid social and outdoor activities, withdraw socially, develop negative attitudes toward treatment, reduce treatment adherence, increase the risk of disease recurrence, and ultimately reduce QoL (16). Therefore, clinical attention should be directed toward screening and intervening in FoP among patients with CHF to mitigate its direct adverse effects on QoL.

The mediating role of psychological resilience between FoP and QoL was confirmed, with an effect of 0.508 (33.22% of the total effect). This indicates an indirect pathway whereby FoP reduces resilience, thereby impairing QoL. Psychological resilience serves as a key protective resource against disease-related stress by helping patients regulate emotions and adapt positively to health challenges (17). When FoP levels are high, excessive worry about disease progression may deplete psychological resources, weaken resilience, hinder active coping, and lead to a negative psychological state, thereby reducing QoL. In contrast, patients with higher resilience tend to adopt positive perspectives toward their illness and engage in effective coping strategies, such as seeking medical care, participating in rehabilitation, and obtaining social support, which helps mitigate the impact of the disease and maintain a better QoL (18). These findings are consistent with studies on patients with cancer, diabetes, and other chronic conditions (19). Clinically, targeted interventions, such as resilience training, cognitive restructuring, and peer support, may enhance patients’ ability to cope with disease-related stress and interrupt the indirect pathway between FoP and QoL.

Psychological distress was also identified as an independent mediator, with an indirect effect of 0.344 (22.50% of the total effect). This suggests that FoP influences QoL by increasing psychological distress. As a core psychological stressor in patients with CHF, FoP may directly induce anxiety, depression, and stress. Persistent negative emotions resulting from excessive concern about disease progression, mortality risk, and family burden may adversely affect QoL through psychophysiological mechanisms. Chronic psychological distress can activate the hypothalamic–pituitary–adrenal axis, increase sympathetic activity, elevate heart rate and blood pressure, increase myocardial oxygen consumption, and further impair cardiac function, thereby reducing physical functioning (20). Moreover, psychological distress may weaken patients’ confidence in treatment and self-management, reduce engagement in rehabilitation and social activities, decrease treatment adherence, and lead to recurrent illness, ultimately diminishing QoL.

A serial mediation effect involving psychological resilience and psychological distress was also identified, with an effect size of 0.048 (3.14% of the total effect). This indicates a pathway in which FoP reduces psychological resilience, which in turn exacerbates psychological distress, ultimately leading to a poorer QoL. As a stressor, FoP may deplete positive psychological resources (i.e., resilience). Reduced resilience impairs emotional regulation and coping capacity, making patients more vulnerable to psychological problems such as anxiety and depression (21). Psychological distress, in turn, affects cardiac function through psychophysiological mechanisms, reduces self-management ability and treatment adherence, and leads to a poorer QoL (22).

To translate these findings into clinical practice, a multifaceted stepped-care model is proposed: (1) strengthen FoP screening by using the FoP-Q scale for routine assessment of hospitalized and outpatient CHF patients, identify those with moderate or higher FoP levels, establish dedicated records, and implement targeted interventions; (2) enhance psychological resilience by providing resilience training programs, guiding patients in positive coping strategies such as cognitive restructuring, mindfulness-based stress reduction, and emotional regulation, and encouraging support from family and peers; (3) alleviate psychological distress through individualized psychological interventions (e.g., cognitive behavioral therapy and supportive psychotherapy) in collaboration with mental health professionals and pharmacological treatment when necessary; and (4) establish a multidisciplinary team including cardiologists, nurses, psychotherapists, and rehabilitation specialists to provide integrated care encompassing medical treatment, psychological intervention, and rehabilitation guidance, thereby interrupting the pathway of FoP → psychological resilience → psychological distress → QoL, and improving overall patient outcomes.

## 5 Conclusion

This study revealed that patients with CHF manifested considerable FoP and compromised QoL. The relationship was significantly mediated in series by psychological resilience and psychological distress. A dual-impact model was identified: FoP directly and indirectly affected QoL by diminishing psychological resilience and aggravating psychological distress. These findings demonstrate the importance of integrating psychological assessments into standard CHF care. Interventions aimed at enhancing psychological resilience and alleviating psychological distress may mitigate the negative impact of FoP and improve patient well-being.

This study has several limitations that should be acknowledged. First, owing to the inherent limitations of the single-center cross-sectional design, this study can only verify the statistical associations among fear of disease progression (FoP), psychological resilience, psychological distress, and HRQoL in patients with CHF, as well as the goodness of fit of the serial mediation model. However, it cannot establish temporal precedence or causal relationships and cannot rule out reverse causality (e.g., poorer HRQoL may exacerbate FoP). Although the proposed serial mediation pathway is based on stress and coping theory and supported by previous longitudinal evidence in chronic disease populations, cross-sectional data cannot confirm the directionality of the pathway, “FoP → reduced psychological resilience → increased psychological distress → impaired HRQoL.” Future studies should adopt prospective longitudinal designs with multiple follow-up time points (e.g., baseline, 6, 12, and 24 months), apply cross-lagged panel models (CLPM) or random intercept cross-lagged panel models (RI-CLPM), and conduct randomized controlled trials of psychological interventions to verify causal pathways and provide higher-level evidence. Second, the generalizability and external validity of the findings are limited by the sample characteristics. All 212 participants were recruited from a single tertiary grade A hospital in Henan Province, China, and did not include CHF patients from outpatient settings, community healthcare centers, primary care institutions, or other regions with different socioeconomic contexts. Differences in disease severity, treatment status, psychological condition, social support, and HRQoL between inpatient and non-inpatient populations may limit the applicability of the findings. Although the sample size meets the requirements for serial mediation analysis, it may be insufficient for more complex analyses, such as moderated mediation or subgroup analyses. Future research should involve multi-center, multi-regional studies with larger and more diverse samples (e.g., 500–1,000 participants) to enhance representativeness and external validity. Third, this study only examined the serial mediating roles of psychological resilience and psychological distress and controlled for a limited number of covariates (age, disease duration, and NYHA classification). Other potential mediating, moderating, and confounding variables were not included. Previous studies have shown that factors such as self-management ability, coping style, perceived social support, treatment adherence, and sleep quality may also mediate the relationship between disease-related stress and HRQoL. Additionally, variables such as gender, educational level, social support, LVEF, and comorbidity burden may act as moderators, while factors such as income level, insurance type, and prior psychological intervention may confound the observed associations. Future studies should construct more comprehensive moderated serial mediation models incorporating these variables to better elucidate the complex mechanisms underlying the relationship between FoP and HRQoL and to identify precise intervention targets. Finally, all data were collected using validated self-report instruments, which may introduce common method bias. Although measures such as anonymous surveys, standardized procedures, and on-site quality control were implemented to minimize bias, self-reported data remain subject to recall bias and social desirability bias. Future studies should incorporate objective clinical indicators (e.g., LVEF, 6-minute walk distance, rehospitalization rates, and all-cause mortality) and multi-informant assessments (e.g., family and clinician evaluations) to enhance the reliability and comprehensiveness of the findings.

## Data Availability

The original contributions presented in the study are included in the article/supplementary material. Further inquiries can be directed to the corresponding author.
